# MEIS1 Is a Common Transcription Repressor of the miR-23a and NORHA Axis in Granulosa Cells

**DOI:** 10.3390/ijms24043589

**Published:** 2023-02-10

**Authors:** Siqi Wang, Yang Wang, Yibo Chen, Yuqi Li, Xing Du, Yinxia Li, Qifa Li

**Affiliations:** College of Animal Science and Technology, Nanjing Agricultural University, Nanjing 210095, China

**Keywords:** MEIS1, miR-23a/NORHA axis, FoxO1, GC apoptosis, small regulatory network

## Abstract

MicroRNA-23a (miR-23a) is an endogenous small activating RNA (saRNA) involved in ovarian granulosa cell (GC) apoptosis and sow fertility by activating lncRNA NORHA transcription. Here, we reported that both miR-23a and NORHA were repressed by a common transcription factor MEIS1, which forms a small network regulating sow GC apoptosis. We characterized the pig miR-23a core promoter, and the putative binding sites of 26 common transcription factors were detected in the core promoters of both miR-23a and NORHA. Of them, transcription factor MEIS1 expression was the highest in the ovary, and widely distributed in various ovarian cells, including GCs. Functionally, MEIS1 is involved in follicular atresia by inhibiting GC apoptosis. Luciferase reporter and ChIP assays showed that transcription factor MEIS1 represses the transcription activity of miR-23a and NORHA through direct binding to their core promoters. Furthermore, MEIS1 represses miR-23a and NORHA expression in GCs. Additionally, MEIS1 inhibits the expression of FoxO1, a downstream of the miR-23a/NORHA axis, and GC apoptosis by repressing the miR-23a/NORHA axis. Overall, our findings point to MEIS1 as a common transcription repressor of miR-23a and NORHA, and develop the miR-23a/NORHA axis into a small regulatory network regulating GC apoptosis and female fertility.

## 1. Introduction

In organisms, any physiological (e.g., oogenesis), pathological (e.g., tumorigenesis), or even cellular biological process (e.g., cell apoptosis) is governed by multiple genes. Moreover, these genes are not independent of each other and usually form regulatory axes, signaling pathways, and even regulatory networks to work together [1,2]. Interestingly, in addition to co-expression and the same functions, genes of the same regulatory axis or signaling pathway are sometimes directly regulated by the same regulators [3,4]. The C-X-C chemokine ligand 13 (CXCL13) and C-X-C chemokine receptor type 5 (CXCR5) signaling axis, for instance, play an essential role in B cell recruitment and tertiary lymphoid structure formation, which is transcriptionally activated by a common transcription factor RelA, a subunit of the NF-κB family in breast tumors [5]. The transforming growth factor-β (TGF-β) signaling pathway is one of the most important signaling pathways necessary for development and health in mammals, and many of its members have been demonstrated to be the targets of miR-130a-3p, such as TGF-β1 (the main ligand) [6], TGFBR1 (the type I receptor) [7], TGFBR2 (the type II receptor) [8], SMAD4 (only co-SMAD) in esophageal squamous cell carcinoma [9]. Additionally, several direct regulators of the TGF-β signaling pathway, such as SnoN [10] and GCNT [11], are also targets of miR-130a-3p. 

Our previous study showed that an endogenous small activating RNA (saRNA), miR-23a, activates lncRNA NORHA transcription and forms a regulatory axis that controls ovarian granulosa cell (GC) apoptosis and sow fertility [12]. The purpose of this study was to elucidate the common modulators of miR-23a and NORHA axis in porcine GCs to understand the molecular regulation. We identified MEIS1, an important member of the TALE (transcription activator-like effectors) protein family and a transcription factor of key genes related to various physiological and pathological processes [13,14,15,16], as a common transcription factor of the miR-23a and NORHA axis in GCs. Furthermore, we also showed that transcription factor MEIS1 forms a small regulatory network with the miR-23a/NORHA axis by inhibiting their transcription, which further inhibits GC apoptosis. Our findings are of great significance in revealing the new mechanism of follicular atresia and developing potential non-hormone drugs for the treatment of follicular atresia.

## 2. Results

### 2.1. The miR-23a and NORHA Axis Share Potential Common Transcription Factors

**Figure 1:**
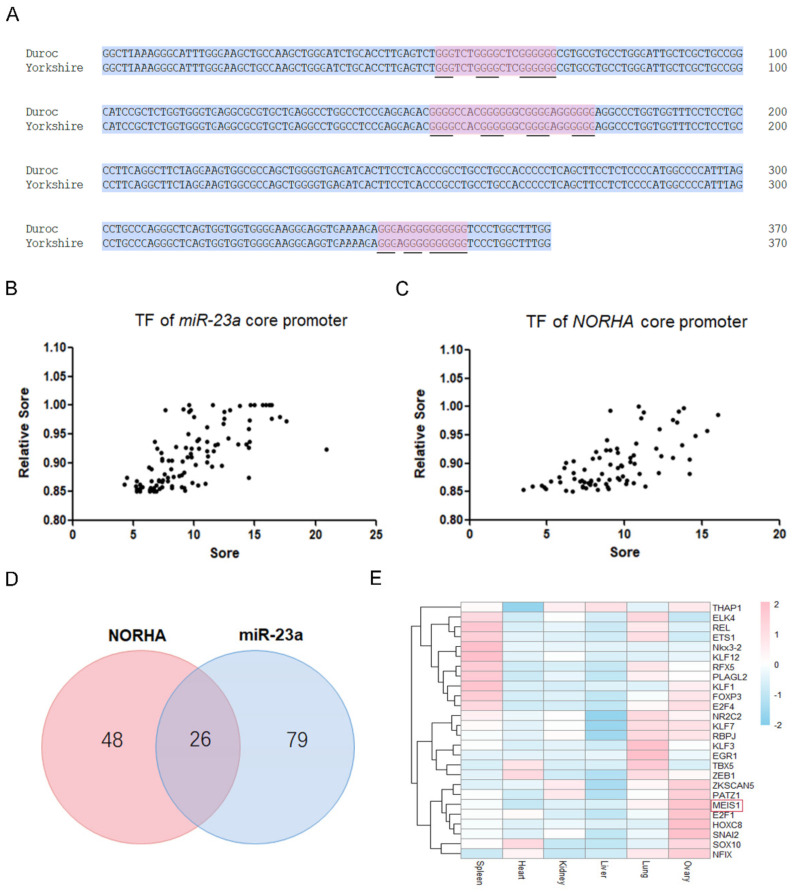
Characterization of the core promoters of the porcine miR-23a and NORHA. (**A**) Sequence analysis of miR-23a core promoter. Blue for consensus sequences and red for G-quadruplexes structures. Underlines indicate the guanine that forms the structure. (**B**,**C**) Prediction of the putative transcription factors interacts with the core promoters of miR-23a (**B**) and NORHA (**C**). The ordinate is the correlation score according to the screened-for binding transcription factors. The abscissa is the binding score according to the strength of binding as a transcription factor. Each dot represents a transcription factor. (**D**) Venn diagram of potential common transcription factors interacts with the core promoters of miR-23a and NORHA. (**E**) Tissue expression profiles of potential common transcription factors interact with the core promoters of miR-23a and NORHA. Expression data were obtained from the GenBank database (https://www.ncbi.nlm.nih.gov/, (accessed on 18 May 2022)).

We previously identified the core promoter of the Yorkshire pig miR-23a gene [12], and here we further characterized this region. The core promoter of Yorkshire pig miR-23a is 370 bp in length, and the consistency with the reference genome sequence (Duroc pig) is 100% (Figure 1A and Figure S1). Binding sites of 105 transcription factors such as MEIS1, THAP1, SMAD4, and NFIX were identified in the core promoter of Yorkshire pig miR-23a (Figure 1B, Table S1). In addition, three DNA G-quadruplexes were found in this region (Figure 1A). However, no miRNA response element (MRE) was detected in this region. Interestingly, the miR-23a core promoter shares 26 potential common transcription factors with the core promoter of Yorkshire pig NORHA (−279 nt~−128 nt), a direct downstream lncRNA of miR-23a in porcine GCs [12] (Figure 1C,D, Table S2). However, DNA G-quadruplexes observed in the miR-23a core promoter were not detected in the NORHA core promoter. However, we discovered an MRE motif of miR-139 in this region (Figure S2). Tissue expression profile showed that several transcription factors including MEIS1, E2F1, HOXC8, and SNAI2 were relatively highly expressed in the ovary of pigs (Figure 1E). These results suggest that miR-23a and NORHA, two components of the miR-23a-NORHA axis may be regulated by the potential common transcription factors in the porcine ovary.

### 2.2. MEIS1 Is a Transcription Factor Related to Sow Follicular Atresia

**Figure 2:**
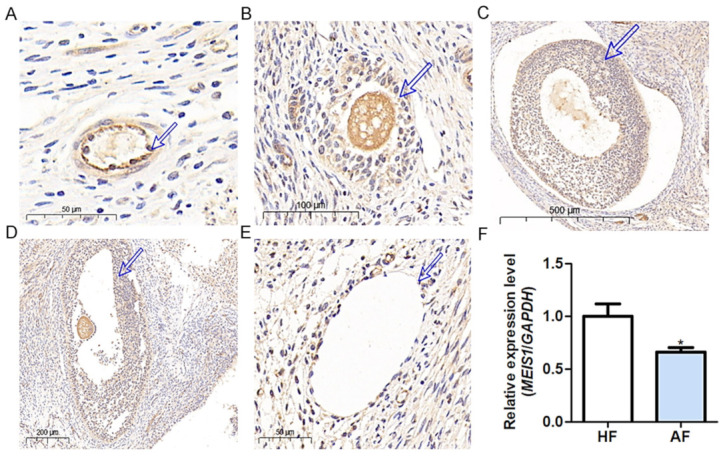
Immunohistochemical assessment for MEIS1 in the ovary during porcine follicular development and atresia. (**A**) Primary follicle. Bar = 50 μm. (**B**) Preantral follicle. Bar = 100 μm. (**C**) Antral follicle. Bar = 500 μm. (**D**) Mature follicle. Bar = 200 μm. (**E**) Atretic follicle. Bar = 50 μm. (**F**) The levels of sow healthy follicles and atretic follicles. HF, healthy follicles. AF, atretic follicles. Arrows indicate GCs. Data are expressed as the mean ± S.E.M, and significance was tested using a *t*-test (* *p* < 0.05).

The expression of MEIS1 was the highest among the above transcription factors highly expressed in the porcine ovary (Table S3), so MEIS1 was selected for further study. Immunohistochemical (IHC) assay with the MEIS1-specific antibody showed that it was observed in sow follicles at various stages of development (Figure 2A–F). In primary follicles, MEIS1 was widely expressed in various cells including GCs, theca cells, and oocytes of the porcine ovary, especially in oocytes and GCs with higher the abundance (Figure 2A–D). Notably, compared to healthy follicles, abundance of MEIS1 in atretic follicles was lower (Figure 2E), which was mainly due to the number of GCs. Consistent with this, qPCR showed that MEIS1 levels were significantly higher in healthy follicles than those in atretic follicles (Figure 2F). In general, the above results suggest that the transcription factor MEIS1 may be related to the follicular development and atresia in the sow ovary.

### 2.3. MEIS1 Is a Novel Suppressor of Apoptosis in GCs 

**Figure 3:**
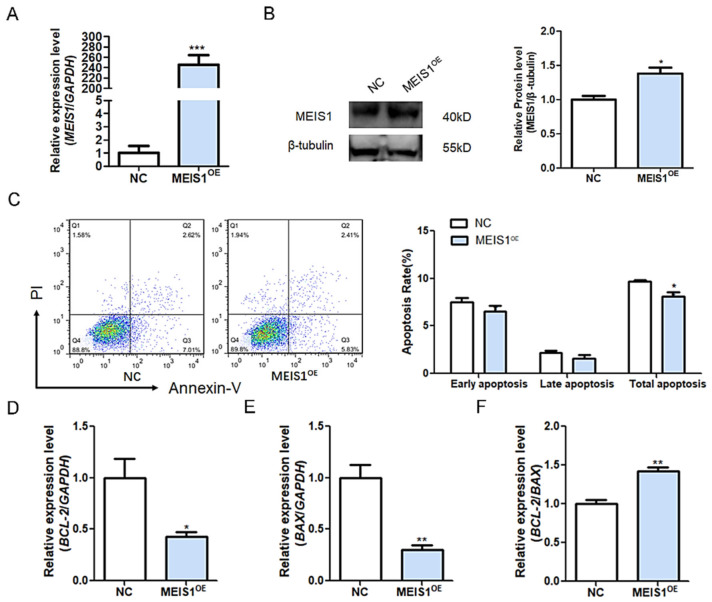
MEIS1 inhibits porcine GC apoptosis. (**A**,**B**) The mRNA (**A**) and protein (**B**) levels of MEIS1 in GCs after transfection of overexpression plasmid pcDNA3.1-MEIS1. (**C**) The apoptosis rate of GCs was determined by flow cytometry after transfection with pcDNA3.1-MEIS1 at 48 h. Different colors indicate different cell densities followed by red, green, and blue. (**D**–**F**) The levels of BCL-2 (**B**) and BAX (**C**) in GCs after common transfection with pcDNA3.1-MEIS1 and miR-23a mimics at 48 h, and the BCL-2/BAX ratio was analyzed (**D**). Data are expressed as the mean ± S.E.M, and significance was tested using a *t*-test (* *p* < 0.05, ** *p* < 0.01, *** *p* < 0.001). NC, negative control.

It is well known that sow follicular atresia is trigged by GC apoptosis. We, therefore, examined the role of MEIS1 in the apoptosis of porcine GCs. The expression of MEIS1 in porcine GCs cultured *in vitro* was significantly over-expressed after transfection with the constructed plasmid pcDNA3.1-MEIS1 (Figure 3A,B). Flow cytometry revealed that overexpression of MEIS1 significantly decreased the total apoptosis rate of GCs, not the early and late apoptosis (Figure 3C). In addition, apoptosis-related genes BCL-2 (Figure 3D) and BAX (Figure 3E) mRNA levels were significantly decreased, and the BCL-2/BAX ratio (Figure 3F) was significantly increased in GCs transfected with pcDNA3.1-MEIS1 compared with the control group. Taken together, these observations above indicate that transcription factor MEIS1 inhibits porcine GC apoptosis.

### 2.4. MEIS1 Inhibits miR-23a Transcription Activity by Binding to Its Core Promoter

**Figure 4:**
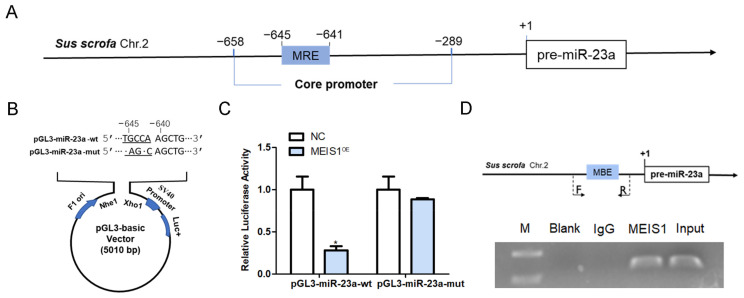
MEIS1 represses the transcriptional activity of the miR-23a core promoter. (**A**) Schematic showing the binding site of transcription factor MEIS1 in the miR-23a core promoter. (**B**) Schematic diagram of the wild-type (wt) and mutated-type (mut) MBE motif in the miR-23a core promoter. Dotted section for MBE. F1 ori, SV40 promoter, and Luc+ indicate important elements on the pGL3 vector. (**C**) Effects of MEIS1 on the activity of reporter vectors pGL3-miR-23a-wt and pGL3-miR-23a-mut. (**D**) ChIP assay. Top: schematic diagram of primers for ChIP assay. Bottom: ChIP image. F/R, the positions of the primers used to amplify a DNA fragment containing the MBE motif. F is the upstream primer and R is the downstream primer. Data are expressed as the mean ± S.E.M, and significance was tested using a *t*-test (* *p* < 0.05). NC, negative control.

To verify whether MEIS1 is a common transcription factor of both miR-23a and NORHA in pigs, we first investigated the regulatory effect of MEIS1 on the transcriptional activity of the miR-23a core promoter. An MEIS1-binding element (MBE) motif (5′-TGACA-3′) was found at −645 nt~−641 nt of the core promoter of the porcine miR-23a gene (Figure 4A). We next constructed two reporter vectors for miR-23a core promoter containing wild-type (wt) or mutated-type (mut) MBE motif, respectively (Figure 4B), and commonly transfected with MEIS1 overexpression vector pcDNA3.1-MEIS1 into porcine GCs. Luciferase reporter assays showed that the luciferase activity of pGL3-miR-23a-wt, but not pGL3-miR-23a-mut, was significantly decreased by transcription factor MEIS1 in GCs (Figure 4C). ChIP assay further confirmed that MEIS1 was able to bind to the MBE motif in the miR-23a core promoter in porcine GCs (Figure 4D). These data suggest that MEIS1 acts as a transcription factor to repress the transcriptional activity of miR-23a core promoter in porcine GCs.

### 2.5. MEIS1 Inhibits NORHA Transcription Activity by Binding to Its Core Promoter

**Figure 5:**
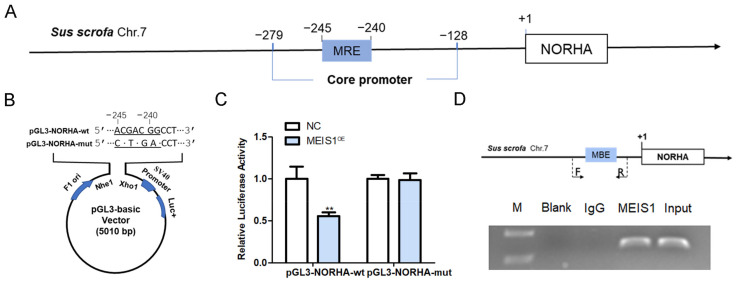
MEIS1 represses the transcriptional activity of the NORHA core promoter. (**A**) Schematic diagram of MBE position in the NORHA core promoter. (**B**) Schematic diagram of reporter vectors of NORHA core promoter with the MBE-wt and MBE-mut motif. Dotted section for MBE. F1 ori, SV40 promoter, and Luc+ indicate important elements on the pGL3 vector. (**C**) Effects of MEIS1 on the activity of reporter vectors pGL3-NORHA-wt and pGL3-NORHA-mut. (**D**) ChIP assay. Top: schematic diagram of primers for ChIP assay. Bottom: ChIP image. F/R, the positions of the primers used to amplify DNA fragment containing the MBE motif. F is the upstream primer and R is the downstream primer. Data are expressed as the mean ± S.E.M, and significance was tested using a *t*-test (** *p* < 0.01). NC, negative control.

We next verified whether the regulatory effects of transcription factor MEIS1 on the transcriptional activity of the NORHA core promoter. An MBE site (5′-CTGTCA-3′) was discovered at −245 nt~−239 nt of the core promoter of the porcine NORHA gene (Figure 5A). We next constructed two reporter vectors with NORHA core promoter containing MBE-wt and MBE-mut motif (Figure 5B), and commonly transfected with pcDNA3.1-MEIS1 into porcine GCs. The luciferase activity of pGL3-NORHA-wt, but not pGL3-NORHA-mut, was significantly decreased after MEIS1 overexpression in porcine GCs (Figure 5C). ChIP assay further confirmed that transcription factor MEIS1 was able to bind to the MBE motif in the NORHA core promoter in porcine GCs (Figure 5D). These results suggest that MEIS1 acts as a transcription factor and inhibits the transcriptional activity of NORHA core promoter in porcine GCs.

### 2.6. MEIS1 Commonly Transcriptional Represses the miR-23a/NORHA Axis in GCs

**Figure 6:**
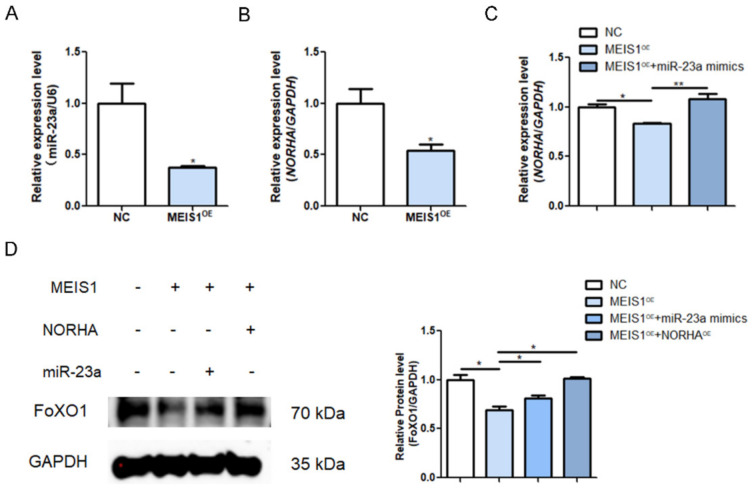
MEIS1 transcriptional represses the miR-23a/NORHA axis in porcine GCs. (**A**,**B**) miR-23a (**A**) and NORHA (**B**) levels were detected in GCs after transfection with pcDNA3.1-MEIS1 at 48 h. (**C**) NORHA levels in GCs after common transfection with pcDNA3.1-MEIS1 and miR-23a mimics at 48 h. (**D**) FoxO1 protein levels in GCs after common transfection with pcDNA3.1-MEIS1 and miR-23a mimics, or pcDNA3.1-NORHA. Data are expressed as the mean ± S.E.M, and significance was tested using a *t*-test (**B**) and one-way ANOVA (**C**,**D**) (* *p* < 0.05, ** *p* < 0.01). NC, negative control.

To demonstrate whether MEIS1 regulates endogenous miR-23a and NORHA expression, we transfected with the overexpression plasmid pcDNA3.1-MEIS1 into porcine GCs. The qPCR revealed that overexpression of MEIS1 significantly reduced the levels of miR-23a and NORHA in GCs (Figure 6A,B). To test whether MEIS1 is able to regulate NORHA expression indirectly via miR-23a, both the pcDNA3.1-MEIS1 and miR-23a mimics were commonly transfected into GCs. The overexpression of miR-23a significantly reversed the decrease in the NORHA expression due to the overexpression of MEIS1 as shown by qPCR (Figure 6C). In addition, a common transfection experiment with pcDNA3.1-MEIS1 and miR-23a mimics or pcDNA3.1-NORHA showed that MEIS1 significantly suppresses the expression of FoxO1, a downstream target of the miR-23a/NORHA axis, while the overexpression miR-23a or NORHA can reverse the condition in GCs, respectively (Figure 6D). In summary, these data suggest that transcription factor MEIS1 commonly transcriptional represses the miR-23a/NORHA axis in porcine GCs.

### 2.7. MEIS Inhibits GC Apoptosis through Inhibiting the miR-23a/NORHA Axis

**Figure 7:**
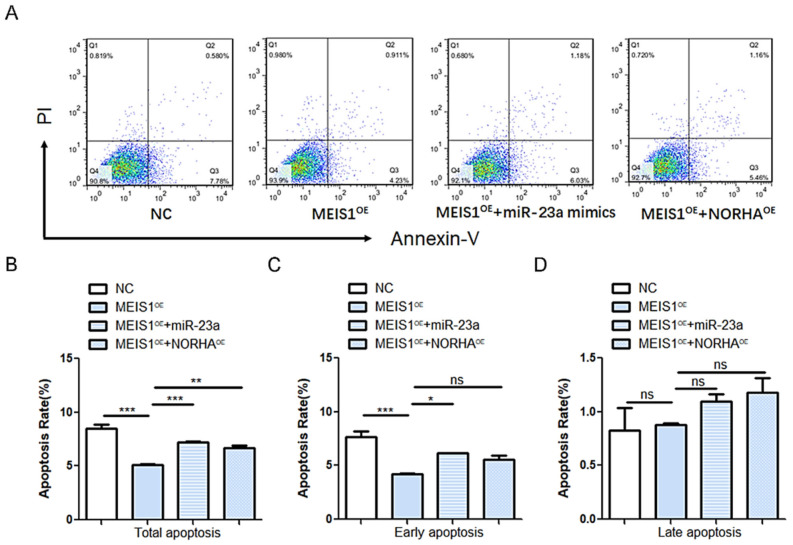
MEIS1 inhibits apoptosis of porcine GCs by repressing the miR-23a/NORHA axis. (**A**) Diagram of flow cytometry. Different colors indicate different cell densities followed by red, green, and blue. (**B**–**D**) Apoptosis rates. Early (Quadrant 4) (**B**), late (Quadrant 2) (**C**), and total (Sum of quadrants 2 and 4) (**D**) apoptosis rates were examined in GCs after common transfection of pcDNA-3.1 MEIS1 and miR-23a mimics or pcDNA3,1-NORHA at 48 h. The apoptosis rate is the ratio of apoptotic cells to total cells. Data are expressed as the mean ± S.E.M, and significance was tested using one-way ANOVA (ns, *p* > 0.05; * *p* < 0.05, ** *p* < 0.01, *** *p* < 0.001). NC, negative control.

To further test whether transcription factor MEIS1 inhibits porcine GC apoptosis via the miR-23a/NORHA axis, common transfected experiments with pcDNA3.1-MEIS1 and miR-23a mimics or pcDNA3.1-NORHA were carried out in porcine GCs. Flow cytometry displayed that overexpression of miR-23a reversed the decreased total apoptotic rate of GCs caused by MEIS1 overexpression (Figure 7A,B). Similarly, overexpression of NORHA also reversed the decreased total apoptotic rate of GCs caused by MEIS1 overexpression (Figure 7A,B). Interestingly, miR-23a was also able to significantly reverse the down-regulation of the early apoptotic rate of porcine GCs caused by MEIS1 (Figure 7C,D). Together, these results suggest that transcription factor MEIS1 inhibits porcine GC apoptosis by suppressing the miR-23a/NORHA axis.

## 3. Discussion

In sows, more than 99% of follicles will undergo atresia during development, and these atretic follicles will fail to ovulate and fertilize, causing waste of follicular resources, which has become one of the important factors hindering sow fertility improvement [17]. Therefore, exploring the molecular regulatory mechanisms of follicular atresia has always been an important research focus for rescuing follicular atresia, improving follicle utilization, and sow fertility. Compelling evidence implicates that sow follicular atresia is also a process governed by multiple genes, and dozens of functional genes including protein-coding genes and non-coding RNAs (ncRNAs) have been identified [12,18,19]. These functional genes usually do not work alone, but rather control follicular atresia by forming signaling axes (e.g., USP9X-TGFBR2 axis), signaling pathways (e.g., NORHA/miR-183C/FoxO1) or molecular regulatory networks (e.g., crosstalk network between TGF-β and Wnt signaling pathways) [20,21,22]. Recently, our study has reported that miR-23a and NORHA, a lncRNA involved in follicular atresia, form a signaling axis through an RNA activation mechanism and induce porcine GC apoptosis through enhancing FoxO1 [12]. Here, we showed that transcription factor MEIS1 is a common transcriptional repressor of miR-23a and NORHA, and represses the activation of the miR-23a/NORHA axis and its mediated GC function. Interestingly, our previous study showed that “transcriptional mis-regulation” is one of the pathways with significant enrichment of differentially expressed genes during early atresia of sow follicles, and multiple follicular atresia-related transcription factors such as CEBPB, HIF1A, and ID2 were identified [23]. Furthermore, several important functional genes, signaling axes, and signaling pathways involved in sow follicular atresia have been shown to be mediated by transcription factors, such as lncRNA BRE-AS, miR-425/TGFBR2 axis, and TGF-β family signaling pathway in porcine GCs being mediated by transcription factor SMAD4 [24,25]. In summary, our findings not only uncover transcription regulation underlying the miR-23a/NORHA axis, but also concluded that transcription factor-mediated transcription disorder is one of the important mechanisms of sow follicular atresia. 

In female reproduction, MEIS1 was not only confirmed to be an important regulator of embryonic development [26], but also proved to regulate the transcription of multiple ovarian cancer-related chemokines, which is expected to be a positive prognostic indicator for ovarian cancer [27]. In this study, we showed that MEIS1 acts as a key transcription factor to inhibit sow follicular atresia and GC apoptosis. In fact, MEIS1 was presented in human GCs at essentially all stages of follicular development [28]; however, in addition to regulating ovarian cancer [27], its role in the ovary and GCs are not clear. In other cell types, MEIS1 has been proved to be an important regulator of apoptosis, which has emerged as a therapeutic target in oncology [15]. In Ewing sarcoma, for instance, transcription factor MEIS1 was identified as a super-enhancer-associated gene, which co-operates with EWS-FLI1 oncoprotein to directly co-bind super-enhancer regions of APCDD1, and activates the transcription of APCDD1, thereby inhibiting cell apoptosis [29]. A recent study reported that MEIS1 inhibits apoptosis of glioma cells by upregulating GFI1 expression [13]. Additionally, in male mice, Sertoli cell-specific MEIS1 knockdown transgenic individuals show massive germ cell loss and impaired spermatogenesis due to Sertoli cell apoptosis [28]. Taken together, our findings expand the target cell types and of MEIS1, and define a new molecular network composed of a transcription factor MEIS1, a signaling axis miR-23a/NORHA, and an effector molecule FoxO1 to regulate GC apoptosis. 

In view of the core regulatory role of ncRNAs in some signal pathways and molecular regulatory networks controlling important physiological and pathological processes, these RNAs are regarded as molecular targets for the improvement of important economic traits in domestic animals [12,30], even potential markers for tumor diagnosis, and potential non-hormone drugs for disease treatment in humans [31,32]. Interestingly, both MEIS1 and NORHA were discovered to be located within the quantitative trait loci (QTLs) for sow fertility traits [33,34], a class of economically important traits in pig production. This is combined with our previous finding (confirming that miR-23a is a causal gene of sow fertility traits [12]), suggesting that MEIS1-miR-23a-NORHA network is a potential network for sow fertility traits and that miR-23a and NORHA, two ncRNAs in this network, can be used as molecular targets for improvement of sow fertility traits. Meanwhile, inhibitors of miR-23a and NORHA also served as the potential non-hormonal RNA drugs for the treatment of sow follicular atresia, considering that miR-23a and NORHA are both strongly involved in follicular atresia [12,22], and demonstrated here to mediate follicular atresia-related transcription factor MEIS1 to control GC apoptosis. Notably, three MBE motifs were discovered in the promoter region of human miR-23a (Figure S3), and miR-23a is highly expressed in patients with colorectal cancer (CRC), whereas MEIS1 is lowly expressed [35,36]. Importantly, diminished MEIS1 expression and raised miR-23a expression harm the survival of CRC patients, which suggested their potential as therapeutic targets [36,37,38]. In addition to colorectal cancer, as a core component of the key signaling pathways and networks that regulate the occurrence of diseases including cancers, miR-23a is considered to be a marker for diagnosis and a potential drug for the treatment of a variety of diseases including cancers, such as glioma [39,40], lung cancer [41], mast cell proliferative disorders [42], and Parkinson’s disease [43,44]. However, for sow miR-23a and NORHA, further studies and practices are needed to better evaluate the application their value in pig production.

## 4. Materials and Methods

### 4.1. Bioinformatic Analysis

The core promoter sequences of miR-23a (chr 2: 65307449-65307818) and NORHA (chr 7: 100139559-100139710) in Duroc pig were downloaded from the GenBank database (https://www.ncbi.nlm.nih.gov/ (accessed on 12 May 2022)). The binding sites of transcription factors in the promoter were predicted by JASPAR (http://jaspar.genereg.net/ (accessed on 15 May 2022)), relative sore > 85%). Guanine (G)-rich four-stranded helical nucleic acid structures (G-quadruplexes) were predicted using an online tool (https://pqsfinder.fi.muni.cz/ (accessed on 23 May 2022)). The expression data of transcription factors were downloaded from NCBI database (https://www.ncbi.nlm.nih.gov/gds/?term=GSE77776 (accessed on 18 May 2022)) and Iswine database (http://iswine.iomics.pro/pig-iqgs/iqgs/index (accessed on 18 May 2022)).

### 4.2. Immunohistochemistry (IHC)

Fresh ovaries were collected from adult sows and immediately immersed in 4% paraformaldehyde for fixation. After 24 h, the samples were sent to Servicebio (Wuhan, China) for embedding, sectioning, and staining. The rabbit anti-MEIS1 antibody (A18273, ABclonal, Wuhan, China) was used for slice incubation, and hematoxylin (Sigma, Shanghai, China) was used to counterstain the nucleus.

### 4.3. Plasmid Construction and Oligonucleotide Synthesis

For reporter construction, DNA fragments of miR-23a or NORHA promoter with MBE motif were cloned into the pGL3-basic vector (Promega, Madison, USA) and verified by DNA sequencing. Overexpression plasmids pcDNA3.1-MEIS1 and pcDNA3.1-NORHA for pigs were previously constructed and preserved in our laboratory. Primers for constructs are shown in Table S4. Oligonucleotides were synthesized by Tsingke (Nanjing, China) according to the following sequence: 5′-AUC ACA UUG CCA GGG AUU UCC-3′ for miR-23a mimics, and 5′-UUG UAC UAC ACA AAA GUA CUG-3′ for mimics NC (Scrambled sequences were immediately followed as a negative control.). The pGL3-miR-23a-mut and pGL3-NORHA-mut were constructed by Tsingke (Nanjing, China). 

### 4.4. Cell Culture, Transfection, and Luciferase Reporter Assays

GCs were isolated and cultured using previously described methods [12]. Briefly, fresh ovaries from adult sows were washed alternately with saline and 75% alcohol, and follicular fluid from healthy follicles was drawn, collected into centrifuge tubes, and centrifuged to collect cells. After washing with phosphate-buffered saline, the cells were resuspended using DMEM/F12 medium (containing 15% fetal bovine serum and 1% penicillin), seeded into cell culture plates, and incubated at 37 °C in a 5% CO_2_ incubator. When both GC density and status reached the transfection requirement, plasmids (1000 ng per well) were transfected into GCs by using HighGene (ABclonal, Wuhan, China) according to instructions. After 24 h of transfection, GCs (5 × 10^5^) were collected for luciferase reporter assay. Luciferase activity was measured using a luciferase reporter assay kit (Vazyme, Nanjing, China) according to the instructions.

### 4.5. Chromatin Immunoprecipitation (ChIP)

The 2 × 10^6^ cells were treated with paraformaldehyde (37%) to crosslink chromatin to proteins, and harvested and lysed after terminating crosslinking using glycine. Then, the reaction was blocked by ultrasonication, and protein-DNA complexes were pulled down using 5 μL anti-MEIS1 antibody (A18273, Abclonal, Wuhan, China). After de-crosslinking, DNA fragments were detected for enrichment by PCR. Antibody against IgG (D110058, Biotechnology, Shanghai, China) was taken as the internal control, and unprocessed chromatin was taken as the Input control. Primers for ChIP are shown in Table S5.

### 4.6. RNA Extraction and Quantitative Real-Time PCR (qPCR)

Total RNA was drawn from GCs using Trizol (Vazyme, Nanjing, China), and reverse transcribed into cDNA using HiScript IIQ Select RT SuperMix (Vazyme, Nanjing, China). Then, qPCR was performed on StepOnePlus System (Applied Biosystems, Waltham, USA) for the same concentration of cDNA using the SYBR Green Master Mix Kit (Vazyme, Nanjing, China). U6 was used as an internal reference for miR-23a, and GAPDH was used as an internal reference for mRNAs and lncRNAs. Finally, the relative expression level of the gene was calculated using 2^−ΔΔCT^ method. Primers for qPCR were shown in Table S6.

### 4.7. Western Blotting

Western blotting was acted as mentioned earlier [12]. The antibodies include anti-FoxO1 (D190665, Sangon Biotech, 1:1000, Wuhan, China) and anti-GAPDH (TA802519, ORIGENE, 1:3000, MD, USA). GAPDH was measured as an internal control. Protein bands were detected with an ultrasensitive chemiluminescent gel imaging system (Bio-Rad, Herclules, USA). Results were analyzed using ImageJ 1.4.3 (NIH, PHILA, USA) for grayscale statistics.

### 4.8. Flow Cytometry

A total of 48 h after transfection, 5 × 10^5^ GCs were collected and stained for 10 min using 5 μL Annexin V (Vazyme, Nanjing, China) and 5 μL Propidium Iodide (PI) (Vazyme, China), a d the apoptosis rate was detected by flow cytometry (Becton Dickinson, Franklin Lakes, USA) and analyzed using FlowJo v7.6 software.

### 4.9. Statistical Analysis

GraphPad Prism v5 software (GraphPad Software, SanDiego, USA) was used for statistical analysis and plotting. *t*-test (two groups) and one-way ANOVA (more than two groups) were used for significance testing.

## 5. Conclusions

**Figure 8:**
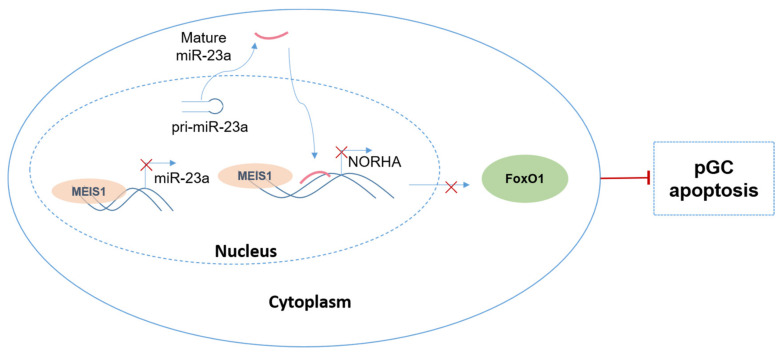
Working model. A novel molecular network controlling GC apoptosis composed of the transcription factor MEIS1, the common inhibitory signaling axis miR-23a/NORHA, and the effector molecule FoxO1.

In conclusion, we identified transcription factor MEIS1 as a common transcription repressor of the miR-23a and NORHA axis in GCs. Furthermore, we also defined a novel molecular network composed of a transcription factor MEIS1, a signaling axis miR-23a/NORHA, and an effector molecule FoxO1 to control GC apoptosis (Figure 8). Overall, our findings provide a new insight into the molecular mechanism of follicular atresia, that is, from a single gene, a signal axis, to a regulatory network.

## Data Availability

No new data were created or analyzed in this study. Data sharing is not applicable to this article.

## Supplementary Materials

The following supporting information can be downloaded at: https://www.mdpi.com/article/10.3390/ijms24043589/s1.

## References

[1] Negrete J.J., Oates A.C. (2021). Towards a physical understanding of developmental patterning. Nat. Rev. Genet..

[2] Vaddavalli P.L., Schumacher B. (2022). The p53 network: Cellular and systemic DNA damage responses in cancer and aging. Trends Genet..

[3] Gao S.H., Liu S.Z., Wang G.Z., Zhou G.B. (2021). CXCL13 in cancer and other diseases: Biological functions, clinical significance, and therapeutic opportunities. Life.

[4] Liu L., Li Q., Yang L., Li Q., Du X. (2021). SMAD4 Feedback activates the canonical TGF-beta family signaling pathways. Int. J. Mol. Sci..

[5] Biswas S., Roy C.S., Mandal G., Purohit S., Gupta A., Bhattacharyya A. (2019). RelA driven co-expression of CXCL13 and CXCR5 is governed by a multifaceted transcriptional program regulating breast cancer progression. Biochim. Biophys. Acta Mol. Basis Dis..

[6] Du X., Wang L., Li Q., Wu W., Shang P., Chamba Y., Pan Z., Li Q. (2020). miR-130a/TGF-beta1 axis is involved in sow fertility by controlling granulosa cell apoptosis. Theriogenology.

[7] Zhu J., Luo Y., Zhao Y., Kong Y., Zheng H., Li Y., Gao B., Ai L., Huang H., Huang J. (2021). circEHBP1 promotes lymphangiogenesis and lymphatic metastasis of bladder cancer via miR-130a-3p/TGFbetaR1/VEGF-D signaling. Mol. Ther..

[8] Ding Y., Hou Y., Liu Y., Yu T., Cui Y., Nie H. (2022). MiR-130a-3p alleviates inflammatory and fibrotic phases of pulmonary fibrosis through proinflammatory factor TNF-alpha and profibrogenic receptor TGF-betaRII. Front. Pharmacol..

[9] Tian X., Fei Q., Du M., Zhu H., Ye J., Qian L., Lu Z., Zhang W., Wang Y., Peng F. (2019). miR-130a-3p regulated TGF-beta1-induced epithelial-mesenchymal transition depends on SMAD4 in EC-1 cells. Cancer Med..

[10] Ai K., Zhu X., Kang Y., Li H., Zhang L. (2020). miR-130a-3p inhibition protects against renal fibrosis in vitro via the TGF-beta1/Smad pathway by targeting SnoN. Exp. Mol. Pathol..

[11] Hu W., Zheng X., Liu J., Zhang M., Liang Y., Song M. (2021). MicroRNA MiR-130a-3p promotes gastric cancer by targeting Glucosaminyl N-acetyl transferase 4 (GCNT4) to regulate the TGF-beta1/SMAD3 pathway. Bioengineered.

[12] Wang S., Li Y., Zeng Q., Yang L., Du X., Li Q. (2022). A mutation in endogenous saRNA miR-23a influences granulosa cells response to oxidative stress. Antioxidants.

[13] Cheng M., Zeng Y., Zhang T., Xu M., Li Z., Wu Y. (2021). Transcription factor ELF1 activates MEIS1 transcription and then regulates the GFI1/FBW7 axis to promote the development of glioma. Mol. Ther. Nucleic Acids.

[14] Luo L., Zhang J., Tang H., Zhai D., Huang D., Ling L., Wang X., Liu T., Zhang Q., Zhang Z. (2020). LncRNA SNORD3A specifically sensitizes breast cancer cells to 5-FU by sponging miR-185-5p to enhance UMPS expression. Cell Death Dis..

[15] Schneider E., Pochert N., Ruess C., MacPhee L., Escano L., Miller C., Krowiorz K., Delsing M.E., Heravi-Moussavi A., Lorzadeh A. (2020). MicroRNA-708 is a novel regulator of the Hoxa9 program in myeloid cells. Leukemia.

[16] Xiang P., Yang X., Escano L., Dhillon I., Schneider E., Clemans-Gibbon J., Wei W., Wong J., Wang S.X., Tam D. (2022). Elucidating the importance and regulation of key enhancers for human MEIS1 expression. Leukemia.

[17] Zhou J., Peng X., Mei S. (2019). Autophagy in ovarian follicular development and atresia. Int. J. Biol. Sci..

[18] Meng L., Zhao K., Wang C.C., Tao J., Wu Z., Teerds K., Zhang S. (2021). characterization of long non-coding RNA profiles in porcine granulosa cells of healthy and atretic antral follicles: Implications for a potential role in apoptosis. Int. J. Mol. Sci..

[19] Wang H., Zhang Y., Zhang J., Du X., Li Q., Pan Z. (2022). circSLC41A1 resists porcine granulosa cell apoptosis and follicular atresia by promoting srsf1 through miR-9820-5p sponging. Int. J. Mol. Sci..

[20] Du X., Li Q., Yang L., Liu L., Cao Q., Li Q. (2020). SMAD4 activates Wnt signaling pathway to inhibit granulosa cell apoptosis. Cell Death Dis..

[21] Yang L., Wang S., Pan Z., Du X., Li Q. (2022). TGFBR2 is a novel substrate and indirect transcription target of deubiquitylase USP9X in granulosa cells. J. Cell. Physiol..

[22] Yao W., Pan Z., Du X., Zhang J., Liu H., Li Q. (2021). NORHA, a novel follicular atresia-related lncRNA, promotes porcine granulosa cell apoptosis via the miR-183-96-182 cluster and FoxO1 axis. J. Anim. Sci. Biotechnol..

[23] Zhang J., Liu Y., Yao W., Li Q., Liu H., Pan Z. (2018). Initiation of follicular atresia: Gene networks during early atresia in pig ovaries. Reproduction.

[24] Du X., Pan Z., Li Q., Liu H., Li Q. (2018). SMAD4 feedback regulates the canonical TGF-beta signaling pathway to control granulosa cell apoptosis. Cell Death Dis..

[25] Yao W., Du X., Zhang J., Wang Y., Wang M., Pan Z., Li Q. (2021). SMAD4-induced knockdown of the antisense long noncoding RNA BRE-AS contributes to granulosa cell apoptosis. Mol. Ther. Nucleic Acids.

[26] Hu L., Li H., Huang C.L., Chen H., Zhu G., Qian K. (2014). Regulation of myeloid ecotropic viral integration site 1 and its expression in normal and abnormal endometrium. Fertil. Steril..

[27] Karapetsas A., Tokamani M., Evangelou C., Sandaltzopoulos R. (2018). The homeodomain transcription factor MEIS1 triggers chemokine expression and is involved in CD8+ T-lymphocyte infiltration in early stage ovarian cancer. Mol. Carcinog..

[28] Ota T., Asahina H., Park S.H., Huang Q., Minegishi T., Auersperg N., Leung P.C. (2008). HOX cofactors expression and regulation in the human ovary. Reprod. Biol. Endocrinol..

[29] Huang S., Li X., Zheng H., Si X., Li B., Wei G., Li C., Chen Y., Chen Y., Liao W. (2019). Loss of super-enhancer-regulated circRNA NFIX induces cardiac regeneration after myocardial infarction in adult mice. Circulation.

[30] Du X., Liu L., Li Q., Zhang L., Pan Z., Li Q. (2020). NORFA, long intergenic noncoding RNA, maintains sow fertility by inhibiting granulosa cell death. Commun. Biol..

[31] Piergentili R., Basile G., Nocella C., Carnevale R., Marinelli E., Patrone R., Zaami S. (2022). Using ncRNAs as tools in cancer diagnosis and treatment-the way towards personalized medicine to improve patients’ health. Int. J. Mol. Sci..

[32] Cavaliere A.F., Perelli F., Zaami S., D’Indinosante M., Turrini I., Giusti M., Gullo G., Vizzielli G., Mattei A., Scambia G. (2021). Fertility sparing treatments in endometrial cancer patients: The potential role of the new molecular classification. Int. J. Mol. Sci..

[33] Schneider J.F., Nonneman D.J., Wiedmann R.T., Vallet J.L., Rohrer G.A. (2014). Genomewide association and identification of candidate genes for ovulation rate in swine. J. Anim. Sci..

[34] Bidanel J.P., Rosendo A., Iannuccelli N., Riquet J., Gilbert H., Caritez J.C., Billon Y., Amigues Y., Prunier A., Milan D. (2008). Detection of quantitative trait loci for teat number and female reproductive traits in Meishan x Large White F2 pigs. Animal.

[35] Deng Y.H., Deng Z.H., Hao H., Wu X.L., Gao H., Tang S.H., Tang H. (2018). MicroRNA-23a promotes colorectal cancer cell survival by targeting PDK4. Exp. Cell Res..

[36] Li Y., Gan Y., Liu J., Li J., Zhou Z., Tian R., Sun R., Liu J., Xiao Q., Li Y. (2022). Downregulation of MEIS1 mediated by ELFN1-AS1/EZH2/DNMT3a axis promotes tumorigenesis and oxaliplatin resistance in colorectal cancer. Signal Transduct. Target Ther..

[37] Choi J.H., Jang T.Y., Jeon S.E., Kim J.H., Lee C.J., Yun H.J., Jung J.Y., Park S.Y., Nam J.S. (2021). The Small-Molecule Wnt Inhibitor ICG-001 Efficiently Inhibits Colorectal Cancer Stemness and metastasis by suppressing meis1 expression. Int. J. Mol. Sci..

[38] Lee Y., Kim S.J., Choo J., Heo G., Yoo J.W., Jung Y., Rhee S.H., Im E. (2020). miR-23a-3p is a key regulator of IL-17c-induced tumor angiogenesis in colorectal cancer. Cells.

[39] Yachi K., Tsuda M., Kohsaka S., Wang L., Oda Y., Tanikawa S., Ohba Y., Tanaka S. (2018). miR-23a promotes invasion of glioblastoma via HOXD10-regulated glial-mesenchymal transition. Signal Transduct. Target Ther..

[40] Xiao L., Li X., Mu Z., Zhou J., Zhou P., Xie C., Jiang S. (2020). FTO inhibition enhances the antitumor effect of temozolomide by targeting MYC-miR-155/23a cluster-MXI1 feedback circuit in Glioma. Cancer Res..

[41] Hsu Y.L., Hung J.Y., Chang W.A., Lin Y.S., Pan Y.C., Tsai P.H., Wu C.Y., Kuo P.L. (2017). Hypoxic lung cancer-secreted exosomal miR-23a increased angiogenesis and vascular permeability by targeting prolyl hydroxylase and tight junction protein ZO-1. Oncogene.

[42] Kim D.K., Bandara G., Cho Y.E., Komarow H.D., Donahue D.R., Karim B., Baek M.C., Kim H.M., Metcalfe D.D., Olivera A. (2021). Mastocytosis-derived extracellular vesicles deliver miR-23a and miR-30a into pre-osteoblasts and prevent osteoblastogenesis and bone formation. Nat. Commun..

[43] Barbagallo C., Mostile G., Baglieri G., Giunta F., Luca A., Raciti L., Zappia M., Purrello M., Ragusa M., Nicoletti A. (2020). Specific signatures of serum miRNAs as potential biomarkers to discriminate clinically similar neurodegenerative and Vascular-Related diseases. Cell. Mol. Neurobiol..

[44] Salemi M., Marchese G., Lanza G., Cosentino F., Salluzzo M.G., Schillaci F.A., Ventola G.M., Cordella A., Ravo M., Ferri R. (2022). Role and dysregulation of miRNA in patients with parkinson’s disease. Int. J. Mol. Sci..

