# Supplementary material for: MEIS1 Is a Common Transcription Repressor of the miR-23a and NORHA Axis in Granulosa Cells
Source: Int J Mol Sci. 2023 Feb 10;24(4):3589. doi: 10.3390/ijms24043589 (PMC9959593; doi:10.3390/ijms24043589)
Supplement: Supplementary file 1 [file ijms-24-03589-s001.zip › ijms-2148681-supplementary.pdf]

Supplemental Figures

Figure S1 The base composition of miR-23a core promoter

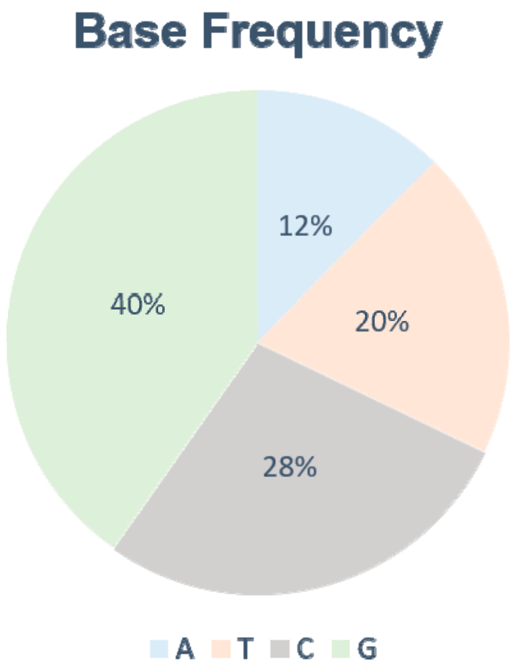

Figure S2 The MRE of NORHA core promoter

AGCCACTCAC TTCCCATCCA GGTCCTTTCA GACGACGGCC 40

TGCATGGTTA CCATGGAGAG GGCCACCCCC CCACCTTTGG 80

GGGCTCTGAG ACCCTAGAAG GAAAGCACCA GGAAACCCCA 120

AAC<u>CGTCTCC</u> CTCCCGAAAA CAATGGCTTC 150

miR-139

Figure S3 The MBE motifs of human miR-23a promoter

```
-500  GCCGGCATCC GCCCTGGTGG GTGTGGGCTA AGCCCTGGCC ACTGAGGAGA CCGGGCCACG

-440  GGGGGGGAGG CCCTGGTGGT TTCCTCCTGC CCTCCAGGCT TCTAGGAAGT GGCGCCAGCT

-380  GGGGTGAGAT CACTTCCTCA CCCGCCTGCC TGGCCCCCCT TGGCTTCCTC TCCCCATGGC

-320  CCCATTTGGC CTGCCCAGGG CTCAATGAGG GGGGAGCTTG GCCATGCAAG TTGCTGTAGC

-260  CTCCTTGTCC CGCATGGGCC CTCTAGGTAT CTCTGCCTCT CCAGTCCTGG GGCTGGAACG
            MEIS1
-200  GAGGGCACAG CTAGGCTCCA GCTCCCCGTG TGGTGGCTCC TGCATATGAG AAAAGAGCTT

-140  CCCTGTGATC AAAGGAAGCA TCTGGGGACC TGGAGGGGAG GTGTCCCCAA ATCTCATTAC
        MEIS1
-80   CTCCTTTGCT CTCTCTCTCT TTCTCCCCTC CAGGTGCCAG CCTCTGGCCC CGCCCGGTGC
                                          MEIS1
-20   CCCCCTCACC CCTGTGCCAC
```

Supplemental Tables

Table S1 Transcription factors in the core promoter of miR-23a

Table S2 Transcription factors in the core promoter of NORHA

Table S3 Expression of common transcription factors for NORHA and miR-23a in porcine ovary.

Table S4 Information of primers for plasmid construction

Table S5 Information of primer for ChIP

Table S6 Information of primers for qPCR

Table S1

Gene	Score
SPI1	1
SP2	1
SP1	1
SP4	1
KLF7	1
KLF14	1
KLF1	1
Ahr::Arnt	1
KLF10	1
KLF12	1
EHF	0.998789
ZEB1	0.993092
Neurod2	0.992556
ETS1	0.992422
Foxn1	0.9923
Olig2	0.988489
Tcfl5	0.988429
NFIX	0.980404
PATZ1	0.979816
BHLHE22	0.977532
Stat4	0.97671
STAT3	0.974285
ZNF281	0.972373
HES1	0.967614
NFIA	0.962395
STAT1	0.958966
ARNT::HIF1A	0.95058
HES2	0.943266
RBPJ	0.941533
TFAP2E	0.938267
Tcf12	0.937483
EGR1	0.936407
NR2C2	0.936261
E2F6	0.932991
TFAP2B	0.932757
NR4A2	0.932736
E2F4	0.9303
THAP1	0.927779
ZKSCAN5	0.926888
ELF5	0.926364
Myog	0.925397
E2F1	0.924473
VAX2	0.924438
PRDM9	0.924095

HES5	0.921013
NR4A1	0.920468
En1	0.917122
MSGN1	0.916828
TFDP1	0.911207
FEV	0.909927
Myb	0.909454
Mafb	0.907874
HIC2	0.904868
Nkx3-2	0.90398
NKX2-8	0.903953
Hic1	0.90249
SREBF2	0.90159
HEY2	0.896944
Egr2	0.894716
ZNF263	0.893573
NR2C1	0.892506
Smad4	0.889715
HOXA7	0.888715
REL	0.883976
MGA	0.880226
PLAGL2	0.879002
ELK4	0.877778
KLF3	0.875281
MEIS1	0.875052
USF1	0.875024
PLAG1	0.874776
Arnt	0.870422
NKX2-4	0.870306
Dlx1	0.868971
TBX4	0.868503
GATA3	0.86833
Mitf	0.867908
Creb3l2	0.866995
CTCFL	0.865057
NFKB1	0.863909
Pax2	0.862972
HOXC8	0.862522
LBX2	0.86168
TBX18	0.86167
NOTO	0.859635
PRDM1	0.85917
TBX5	0.858121
Dlx4	0.857964
NEUROD1	0.857813
Nr1H4	0.857559
NEUROG2	0.857371
HNF4A	0.857197
TBX3	0.857138
ESX1	0.856158

FOXP3	0.855443
NRL	0.855152
ISL2	0.853435
ISX	0.853235
VDR	0.852528
ZNF549	0.852211
SNAI2	0.851357
SOX10	0.85122
TBX6	0.850974
Neurog1	0.850801
RFX5	0.850497

Table S2

Gene	Score
Sox5	1
SOX13	0.997868
ZEB1	0.993092
Sox3	0.992116
SOX18	0.990881
ZNF740	0.986282
SRY	0.97926
VEZF1	0.977241
SOX8	0.972197
SOX4	0.960207
Sox17	0.95815
KLF16	0.948682
FOXD2	0.940945
SP5	0.935939
SP8	0.933059
RFX7	0.926193
SP3	0.925811
Klf4	0.923334
FOXO4	0.923128
FOXP3	0.920351
Foxj2	0.919364
KLF2	0.915262
SP9	0.912395
MSANTD3	0.910746
KLF5	0.909997
Znf423	0.909729
FOXO6	0.908824
RFX3	0.907075
ZNF354C	0.904031
RUNX3	0.903123
HOXC8	0.901077
KLF11	0.899383
HMBOX1	0.897816
ELF5	0.896494
KLF3	0.895369
HOXB6	0.892412
KLF10	0.891671
FOXI1	0.889012
HAND2	0.883764
KLF14	0.883137
RUNX2	0.882618
IKZF1	0.882345
KLF9	0.881577
ZBED2	0.876834

TCF4	0.876256
FOXN3	0.875
THAP1	0.873741
Sox11	0.872208
ELF1	0.871578
NKX2-5	0.870807
FOXG1	0.870697
Zic1::Zic2	0.870243
E2F3	0.8699
TCF3	0.867341
Gli2	0.865601
Nkx3-1	0.865352
ETV5	0.864226
ERF::FOXO1	0.863891
ELF2	0.862577
FEV	0.86198
BSX	0.861154
Wt1	0.860058
HOXA1	0.859851
NFAT5	0.85918
NFYC	0.858701
EGR1	0.858095
NKX2-8	0.858008
KLF6	0.857027
ZBTB26	0.855834
ETS2	0.855255
MEIS1	0.854031
NFYA	0.853494
Erg	0.852506
MAZ	0.851218

Table S3

Transcription factors	Expression value
HOXC8	49.94
E2F1	49.8
SNAI2	50.33
MEIS1	138

The expression value was downloaded from Iswine database (http://iswine.iomics.pro/pig-iqgs/iqgs/index), and the expression value is calculated from RNA-seq data.

Table S4

Name	Primer sequence (5' to 3')	Product size (bp)	Annealing temp (°C)
pGL3-miR-23a	F: CGGGGTACCGGGAAACTGAGGCTTAAAGGG<br>R: CCGCTCGAGCTGCCATCCTTTGAGGTCTTG	558	58
pGL3-NORHA	F: CGGGGTACCAGCCACTCACTTCCCATCCA<br>R: CCGCTCGAGGCCTGTGTTCCTTCCTGTTTC	293	58

Table S5

Name	Primer sequence (5' to 3')	Product size (bp)	Annealing temp (°C)
miR-23a-MRE	F: TTGGTTCCATTTTGCAGATG<br>R: ACCCACCAGAGCGGATG	147	56
NORHA-MBE	F: GCCTTTTAACACCTCCAAG<br>R: TCCTTCTAGGGTCTCAGAGC	150	53

Table S6

Name	Primer sequence (5' to 3')	Product size (bp)	Annealing temp (℃)
miR-23a (NR_038489)	F: CGGGCATCACATTGCCAGG<br>R: CAGCCACAAAAGAGCACAAT	56	60
U6 (EU520423.1)	F: GCTTCGGCAGCACATATACT<br>R: TTCACGAATTTGCGTGTCAT	86	60
NORHA (XR_304632.3)	F: GCTGAACCTGGGGAGTGTCTA<br>R: TCTCTGTCCTTGTGGTGTCTGC	176	60
GAPDH (NM_001206359.1)	F: GGACTCATGACCACGGTCCAT<br>R: TCAGATCCACAACCGACACGT	163	60
stem loop	CCTGTTGTCTCCAGCCACAAAAGAGCAC<br>AATATTTCAGGAGACAACAGGGGAAATC		
